# A novel method for establishing a rat model of knee osteoarthritis: knee joint fixation using medical polyurethane composite wraps

**DOI:** 10.3389/fvets.2026.1725539

**Published:** 2026-04-17

**Authors:** Xiaowen Lian, Maotao Mu, Yaxin Zhuang, Jiehui Fu, Xiumin Jiang, Bingyong Huang, Zexing Guo, Xianglong Feng, Shan Gao, Weiquan Zeng

**Affiliations:** 1 Rehabilitation Hospital Affiliated to Fujian University of Traditional Chinese Medicine, Fuzhou, Fujian, China; 2 Fujian Key Laboratory of Rehabilitation Technology, Fuzhou, Fujian, China; 3 Fujian University of Traditional Chinese Medicine, Fuzhou, Fujian, China; 4 Gutian County Hospital of Traditional Chinese Medicine, Ningde, Fujian, China

**Keywords:** animal model, arthro-braking method, chondrocyte apoptosis, comparative medicine, knee osteoarthritis, veterinary osteoarthritis

## Abstract

**Background:**

Knee osteoarthritis (KOA) is a leading cause of chronic pain and disability in humans, companion and farm animals, with socioeconomic burden and animal welfare concerns. KOA models are essential for advancing diagnostics, therapies, and preventive strategies. This study aimed to establish a reliable non-invasive rat KOA model and evaluate its translational value for comparative and clinical medicine.

**Methods:**

Twenty male Sprague-Dawley rats were randomly allocated to two groups: a model group (*n* = 10) and a control group (*n* = 10). KOA was induced in the model group by immobilizing the knee joint through circular fixation using a medical polyurethane composite bandage. Following the modeling phase, knee cartilage was macroscopically evaluated. Functional impairment was assessed using the Modified Lequesne MG scale. Imaging analyses comprised 7.0 T magnetic resonance imaging (MRI) and micro-computed tomography (Micro-CT) to examine structural and morphological alterations in the knee joint. The histological assessment included Safranin O–Fast Green staining to assess cartilage degradation using the Osteoarthritis Research Society International (OARSI) scoring system. Chondrocyte apoptosis was identified through terminal deoxynucleotidyl transferase dUTP nick end labeling (TUNEL) staining.

**Results:**

The examination of the knee joint cartilage in the model group revealed significant surface irregularities and roughness. Compared to the control group, the model group exhibited a notably higher Modified Lequesne MG score (*P* < 0.01), indicating compromised joint function. Imaging techniques such as high-field MRI and micro-CT confirmed typical osteoarthritic characteristics. Safranin O–Fast Green staining showed a substantial reduction in proteoglycans in the superficial cartilage layer of the model group, along with a significantly increased OARSI score (*P* < 0.01). TUNEL staining indicated a marked rise in chondrocyte apoptosis in the model group, with an approximately 4.8-fold higher rate compared to the control group (*P* < 0.01). The model induction success rate exceeded 95%, with a complication rate below 5%.

**Conclusion:**

We developed a non-invasive rat KOA model using polyurethane bandage immobilization that recapitulates cross-species progressive OA pathology. This robust, low-cost platform enables early biomarker screening, disease-modifying therapy evaluation, and preventive intervention validation for veterinary/human OA, highlighting its translational value for comparative and clinical medicine.

## 1 Introduction

Knee osteoarthritis (KOA), also known as degenerative joint disease, is a chronic, progressive disorder characterized by diffuse articular cartilage degradation, subchondral bone remodeling and sclerosis, osteophyte formation, synovial inflammation and thickening, meniscal degeneration, ultimately resulting in joint pain, restricted mobility, and functional impairment (1). KOA is the most common type of osteoarthritis, predominantly affecting the knee joint and significantly impacting patients' mobility and quality of life.

In 2019, KOA affected an estimated 365 million individuals globally, with an age-standardized prevalence of 7.5% and an incidence of 6.2%, showing an increase compared to 1990 (2). Studies project that by 2050, global cases of KOA will increase by approximately 74.9% compared to 2020 (3). KOA significantly reduces patients' quality of life and poses substantial medical and socioeconomic burdens. Healthcare costs and productivity losses due to osteoarthritis amount to 1–2.5% of Gross domestic product (GDP) in high-income countries, with per-patient annual expenses ranging from thousands to tens of thousands of dollars (4). Importantly, osteoarthritis (including KOA) is also a highly prevalent and burdensome disease in companion and farm animals worldwide (5). It is recognized as one of the most common causes of chronic pain, lameness, and elective euthanasia in companion animals, as well as a significant source of production losses and compromised animal welfare in livestock (6). Critically, KOA in humans, companion animals, and farm animals shares nearly identical core pathological features, disease progression patterns, and unmet clinical needs—including the urgent demand for early diagnostic tools, effective disease-modifying therapies, and targeted preventive strategies (7). While the exact pathogenesis of KOA remains incompletely understood, key risk factors include obesity, high bone mineral density, joint injury, joint instability, joint deformity, and advancing age, particularly affecting weight-bearing joints like the hip and knee (8, 9).

To investigate the pathogenesis of KOA, a robust and consistent animal model is indispensable for preclinical research in both human and veterinary medicine, as human clinical research is strictly limited by ethical constraints, long disease cycle and inability to perform invasive dynamic sampling. Current KOA models can be categorized as either surgical or non-surgical. The conventional Hulth surgical model, while inducing rapid cartilage degeneration, is constrained by significant surgical trauma, infection susceptibility, and inflammation artifacts (10). Non-surgical methods include biochemical induction (e.g., intra-articular collagenase or papain) and arthro-braking techniques (11, 12). Biochemical models are straightforward to execute and replicate, yet they elicit excessive inflammation and deviate from human KOA pathology (13). The Videman arthro-braking model, which immobilizes the knee to simulate degeneration induced by chronic overload, more accurately mirrors the natural progression of the disease (14). However, conventional plaster casts lack adequate fixation strength, are prone to slippage, and pose a risk of ischemic injury, primarily validated in rabbits rather than rats.Existing mainstream preclinical KOA models are generally limited by insufficient stability, variable success rates, and poor fidelity to the natural pathological progression of human KOA, while spontaneous OA models have extremely long induction cycles and poor batch-to-batch consistency (5). These shortcomings underscore the urgent need to develop a simpler, more cost-effective, and highly reproducible KOA model—precisely the objective of the present study. From a comparative medicine perspective, Sprague-Dawley rats exhibit highly conserved knee joint anatomy, cartilage composition, biomechanical properties, and OA pathological progression patterns with humans, companion dogs, and athletic horses (7). The non-invasive immobilization model in this study faithfully recapitulates the progressive course of human clinical OA, providing a solid scientific basis for reliable cross-species data extrapolation. Despite rats being the preferred species for KOA studies due to their affordability, ease of handling, and diverse outcome measures (13). A non-invasive and reliable rat model remains elusive.

Expanding upon these constraints, we have devised a novel, non-invasive rat model of KOA by enhancing the conventional Videman arthro-braking method through upgraded fixation materials and wrapping techniques. Validation of this model was achieved through behavioral assessments, imaging assessments, and histopathological examinations. This dependable model not only enables in-depth investigations into osteoarthritis mechanisms but also provides a sturdy foundation for preclinical assessments of joint mobilization, soft-tissue release, and other rehabilitative strategies, as well as the development of early diagnostic markers, disease-modifying therapies, and preventive interventions for OA applicable to both veterinary and human clinical practice, underscoring its substantial translational promise.

## 2 Materials and methods

### 2.1 Animal management

All experiments were approved by the Animal Experimentation Ethics Committee of Fujian University of Traditional Chinese Medicine (IACUC No. 2023217) and were conducted in accordance with the ARRIVE guidelines. Male Sprague-Dawley rats of SPF grade, weighing 180–200 g and aged 6–7 weeks, were acquired and kept at the Standard Laboratory Animal Center of Fujian University of Traditional Chinese Medicine (Fujian, China). Free access to food and water was provided, and the temperature was maintained between 24 and 26°C. The light environment alternated between 12 and 12 h of light and darkness. After 7 days of adaptation, the rats were separated into two groups: control (*n* = 10) and OA model (*n* = 10) using a computerized random number table approach. On the day of fixation, the control and model groups shaved the right hind limb and sterilized the skin with 70% ethanol in preparation for the arthro-braking.

### 2.2 Main instruments and reagents

Urgo^®^ Elastic Bandage (8 cm × 2.5 m; Shanghai, China; China Medical Device Filing No. 20161621); The medical polymeric fixation bandage was purchased from Zhuhai Jianfan Biotechnology (Zhuhai, China; medical device registration no. Yue Zhu Xie Bei 20150007);304 stainless steel wire (conventional); Bruker BioSpec 70/20 7T scanner (Bruker Biospin, Ettlingen, Germany); Quantum GX2 imaging system (PerkinElmer, Waltham, MA, USA); Safranin O–Fast Green Staining Kit (G1053, Seville Biotechnology, Wuhan, China); MSHOT ML31 (MSHOT Instruments, Guangzhou, China).

### 2.3 KOA model preparation

**Figure 1:**
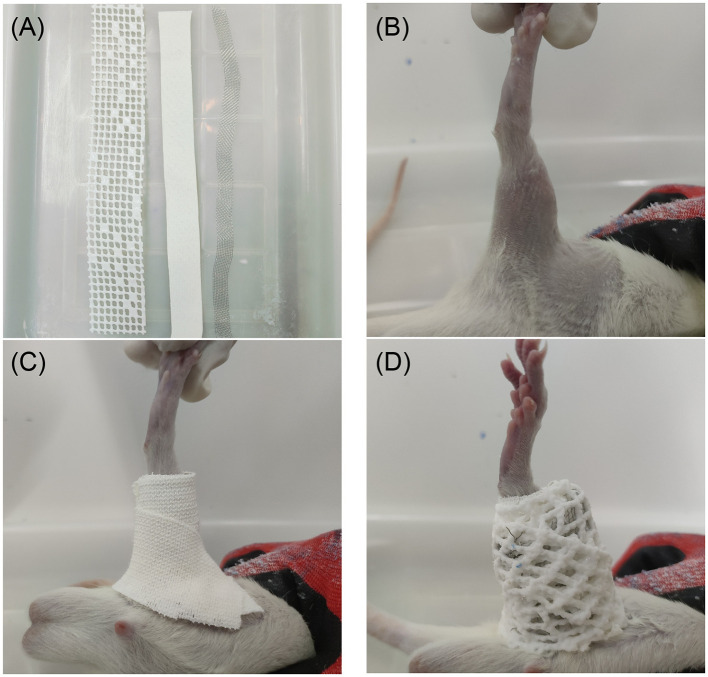
Rat KOA model induction flowchart. **(A)** Non-elastic bandages, 304 stainless steel wire, and bandages made of medical polyurethane-based polymer are the materials needed to make the model. **(B)** The right hind limb's hair removal. **(C)** The non-elastic bandage is wrapped in a spiral pattern around the knee joint at its maximum extension (0°). **(D)** Overlay of the wire-reinforced medical polymeric bandage, with subsequent cooling and hardening to maintain immobilization.

We developed a novel, non-invasive rat KOA model based on the Videman rabbit arthro-braking model (14) (Figure 1). One knee joint was wrapped in a medical polymeric polyurethane composite bandage, which was used to continually stretch and immobilize it for 6 weeks. 304 stainless steel wire (3.0 cm × 2.0 cm), a non-elastic bandage (2.5 cm × 2.0 cm), and a polyurethane-based polymer composite bandage (3.5 cm × 5.0 cm) were vital components. Progressive cartilage deterioration was brought on by constant straightening and load changes, which accurately replicated the pathology of osteoarthritis in the knee.

The rats were first placed in the left lateral decubitus position until they were completely relaxed, and their heads were covered with a soft cotton sleeve to help calm them down. Hair within a 3–4 cm margin around the knee joint was carefully trimmed, and the skin was thoroughly disinfected with 75% medical ethanol—an internationally recognized concentration for skin antisepsis with validated rapid bactericidal efficacy against common cutaneous pathogens, thereby reducing infection risk during the 6-week immobilization period. This concentration is widely used in veterinary clinical practice and laboratory animal operations. Following disinfection, the right hind limb was maintained in full extension (0°). After that, a non-elastic bandage was applied in a spiral pattern from the proximal third of the femur to the ankle, securely enclosing the soft tissues and joint capsule. After that, a medical polyurethane-based polymer composite bandage was heated until it became pliable, a 304 stainless steel wire was inserted in the middle of the bandage along its long axis, and the bandage was wrapped around the underlying layer in a spiral fashion while maintaining full knee extension. After the material cooled and solidified, the immobilization was maintained for 6 weeks, during which time the bandages were checked every day and changed if they were wet or loose. The Modified Lequesne MG scale was used to assess joint function at weeks 2, 4, and 6. Opposite knee swelling, localized hyporesponsiveness, and a total score ≥3 were indicators of successful model induction. This simple, non-invasive procedure consistently causes mechanical stress-driven osteoarthritis in rats' knees, is very reproducible, and has few side effects.

### 2.4 Behavioral assessment

Knee joint dysfunction and pain-related behaviors were evaluated using a rodent-validated Modified Lequesne MG scale (15). Assessments were performed at 5 time points: baseline (before modeling), every 2 weeks for 3 time points during the 6-week modeling period, and after intervention, covering 4 core dimensions: gait pattern, joint swelling, range of motion, and nociceptive response. All tests were conducted in a quiet, temperature-controlled, standardized environment, with 30 min of pre-test acclimation to eliminate stress interference. Two trained independent observers performed all assessments in a double-blinded manner (blinded to rat grouping), and the average score was used for statistical analysis. This scale has a total score range of 0–11 points (higher scores indicate more severe KOA progression and joint impairment), with successful model induction defined as a total score ≥3 at the end of the 6-week modeling period. The core scoring criteria are as follows: (1) “Local pain response (0–3 points)”: Induced by 3 consecutive constant-speed full-range passive flexion-extension of the affected knee, scored by resistance, vocalization and avoidance (0 = no response; 1 = mild resistance; 2 = moderate pain with resistance and vocalization; 3 = severe pain with active avoidance). (2) “Gait and movement pattern (0–3 points)”: Spontaneous gait observed for 5 min in a 100 cm × 100 cm open field, scored by affected limb weight-bearing and lameness (0 = normal full weight-bearing; 1 = mild lameness with slightly reduced weight-bearing; 2 = moderate lameness with limited weight-bearing; 3 = severe lameness with non-weight-bearing limb). (3) “Knee range of motion (0–3 points)”: Maximum flexion-extension angle measured with a protractor under light anesthesia, graded against the contralateral healthy knee (0 = normal range; 1 = < 25% range reduction; 2 = 25%−50% reduction; 3 = >50% reduction or ankylosis). (4) “Joint swelling (0–2 points)”: Knee circumference measured at a fixed anatomical position with a vernier caliper, graded against the contralateral knee (0 = no swelling; 1 = mild swelling < 10% circumference increase; 2 = obvious swelling ≥10% increase).

### 2.5 Combined MRI and micro-CT evaluation

One day following OA induction, rats were sedated with isoflurane (2–3% in O_2_) and positioned supine with their right knee completely extended in a custom-designed fixation cradle. A physiological monitoring system compatible with MRI continually recorded respiration rate and body temperature, guaranteeing steady vital signs and a controlled ambient temperature during the scanning process. A Bruker BioSpec 70/20 USR 7 T small-animal MRI scanner with a rat-specific ^1^H surface receive coil (RF SUC 300, 30 mm ID) and transmit coil (RF RES 300) was used for imaging (*n* = 5 per group). The acquisition parameters were: TR = 940.31 ms, TE = 20.5 ms, flip angle = 90°, slice thickness = 0.5 mm, FOV = 50 × 50 mm, and NEX = 8. All scans were examined independently by two radiologists with experience in osteoarthritis imaging.

Following a 24-h recovery period, rats were re-anesthetized (isoflurane 2%−3% in O_2_) for an *in vivo* Micro-CT of the right knee using a PerkinElmer Quantum GX2 system. The scan settings were 90 kV tube voltage, 80 μA current, and 72 mm FOV. Projection data were reconstructed using the manufacturer's software to create high-resolution 2D cross-sections and 3D images. MRI and micro-CT were employed on the same group of rats.

### 2.6 Tissue preparation

Six weeks later, rats were sedated with an intraperitoneal dose of 3% sodium pentobarbital (30 mg/kg) and euthanized *via* cervical dislocation. The right knee joint was then opened, and the entire femoral condyle cartilage was photographed to compare surface morphology, roughness, and color changes between the control and model groups. Following the removal of adhering tissues, the specimens were preserved in 4% paraformaldehyde at room temperature for 24 h. They were then decalcified in 10% EDTA (pH 7.4) for 6 weeks at 4°C, with the solution renewed every 3 days. Tissues were decalcified, then dehydrated with ethanol, cleaned in xylene, fixed in paraffin, and sectioned longitudinally at 5 μm.

### 2.7 Histology

Paraffin-embedded sections were stained with Safranin O–Fast Green and histopathologically evaluated using the modified OARSI approach for cartilage grading in osteoarthritis [Pritzker et al. (16)]. The grading scale (0–6) represents increasing depth and severity of cartilage damage: 0 = normal, 1 = fine surface fissures, 2 = superficial fissures (< 50% of the thickness), 3 = intermediate fissures (≥50% of the thickness), 4 = basal plate exposed but < 50% of the area, 5 = basal plate exposed and >50% of the area, and 6 = complete cartilage loss with bony plate destruction. Four standardized regions of each histological section were examined under 40 × magnification: the anterior and posterior aspects of the medial femoral condyle, and the anterior and posterior aspects of the medial tibial plateau. The scores of all four regions were summed to obtain the final total OARSI score for each section.

### 2.8 Terminal deoxynucleotidyl transferase dUTP nick end labeling (TUNEL) staining

Chondrocyte apoptosis was measured using a TUNEL apoptosis detection kit (G1507-50T, Xavier Biotechnology, Wuhan, China) following the manufacturer's instructions. Chondrocytes with brown-yellow nuclei were thought to be apoptotic. Each group included three samples. Five fields of view were randomly picked from each sample's cartilage layer, and cell counts and apoptosis rates were estimated with the FIJI software. Apoptosis rate (%) = (number of TUNEL-positive cells / total number of chondrocytes) x 100%.

### 2.9 Statistical analysis

All data are presented as mean ± standard deviation. Differences between two independent groups were evaluated by an unpaired *t*-test when data were normally distributed or by the nonparametric Mann–Whitney U test for non-normal datasets. OARSI scores were compared using the Mann–Whitney U test, whereas apoptosis rates were analyzed with the unpaired *t*-test. Modified Lequesne MG scores across multiple time points were assessed by one-way analysis of variance (ANOVA). All statistical analyses and graphing were performed using GraphPad Prism v10.1.2 (GraphPad Software, San Diego, CA, USA). Two-tailed *P* < 0.05 was considered statistically significant.

## 3 Results

### 3.1 Modified lequesne MG scores

**Figure 2:**
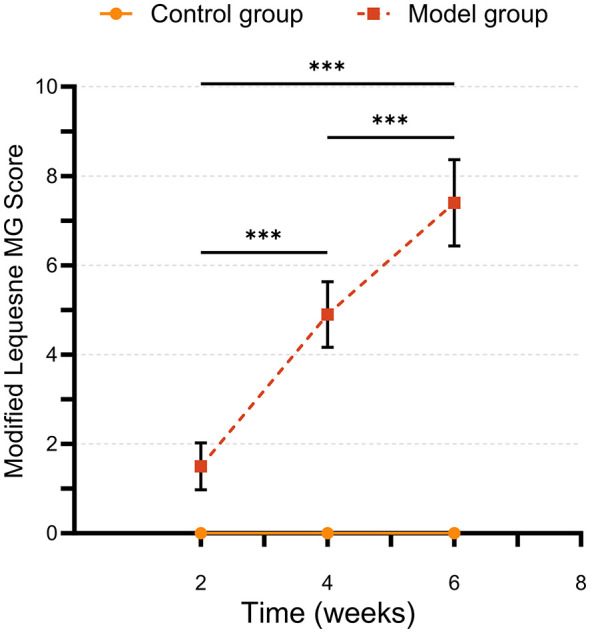
Modified Lequesne MG Scores (mean ± SD, *n* = 10 per group; ***p < 0.001) at weeks 2, 4, and 6 for the control vs. model groups, illustrating a progressive decline in joint function in KOA rats compared to controls. The scores were obtained using a rodent-specific Modified Lequesne MG scale (total score range: 0–11 points, higher score indicates more severe joint dysfunction).

As early as 2 weeks after modeling, the model group's Modified Lequesne MG scores clearly showed early joint dysfunction, being considerably greater than the control group's. The model-group scores increased over time, and at weeks 2, 4, and 6 (*P* < 0.001), the differences were highly significant (Figure 2). In this KOA rat model, these findings show a steady decline in joint function and validate that the Modified Lequesne MG score accurately and sensitively captures the degree of damage.

### 3.2 Macroscopic observation of articular cartilage

**Figure 3:**
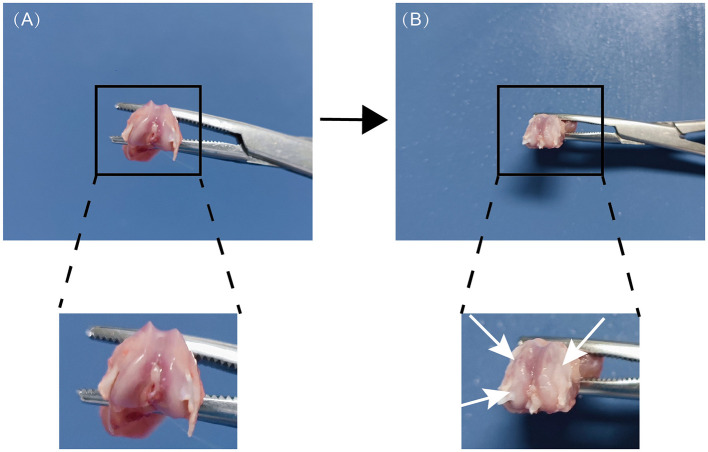
Macroscopic appearance of knee cartilage specimens from control and immobilized groups following model induction. **(A)** Control group: the articular cartilage surface is smooth, glossy, and intact. **(B)** Model group: the articular cartilage exhibits desiccation and surface roughening (white arrows), with osteophyte development at joint borders.

Throughout the trial, all rats consumed similar amounts of food and water while exhibiting similar behavior and activity. Gross inspection revealed that the control group's articular cartilage was smooth, shiny, and well-organized (Figure 3A). In contrast, the model group showed a significant loss of cartilage sheen, with uneven, rough surfaces, focal flaws, and areas of cartilage exfoliation; the joint surface also seemed dehydrated. The prepatellar synovium in these animals was noticeably thicker and yellower (Figure 3B). These macroscopic changes demonstrate obvious indications of joint damage and validate that the KOA model was constructed successfully.

### 3.3 MRI/micro-CT imaging findings

**Figure 4:**
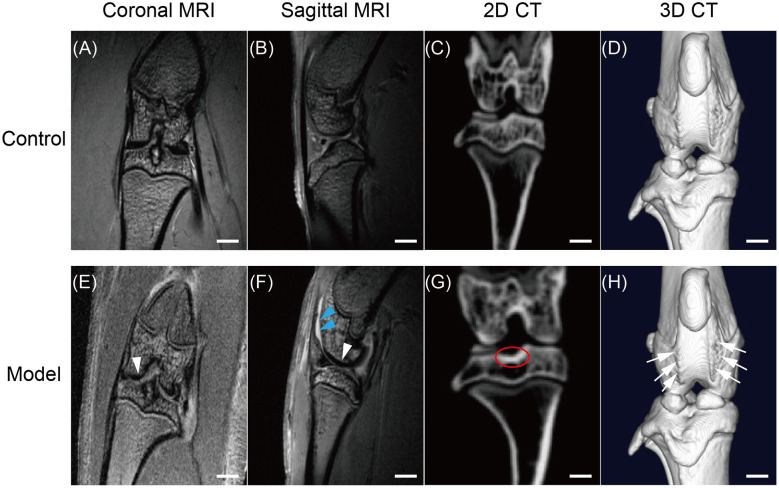
Radiographic comparison of knee joints in control and KOA rats. **(A, B, E**, **F)**: Coronal and sagittal 7T MRI images, respectively, showing medial joint space narrowing [**(E, F)**, white arrows], focal femoral condyle cartilage defects (**E, F**, white arrows), and joint effusion [**(F)**, blue arrows] in the KOA group. **(C, D, G, H)**: μCT 3D reconstructions revealing reduced joint space, marginal osteophyte formation, and subchondral sclerosis beneath the tibial plateau [**(G)**, circled]. Three-dimensional views highlight irregular bone surfaces, patellar malformation, and early osteophyte development around the joint in the KOA group **(H)**, arrows. Scale bar: 5 μm.

During the trial, we used MRI and micro-CT to image the knee joints of KOA and control rats. The MRI revealed a significant narrowing of the tibiofemoral joint space, increased joint effusion, and heterogeneous, irregular signals along the femoral condylar cartilage surface (Figures 4A, B, E, F). Corresponding micro-CT reconstructions revealed joint space constriction, marginal osteophyte formation, and subchondral sclerosis beneath the tibial plateau, as well as prominent surface abnormalities (Figures 4C, D, G, H). The consistent results across both modalities support the accuracy and usefulness of our KOA model.

### 3.4 Histological evaluation of cartilage

**Figure 5:**
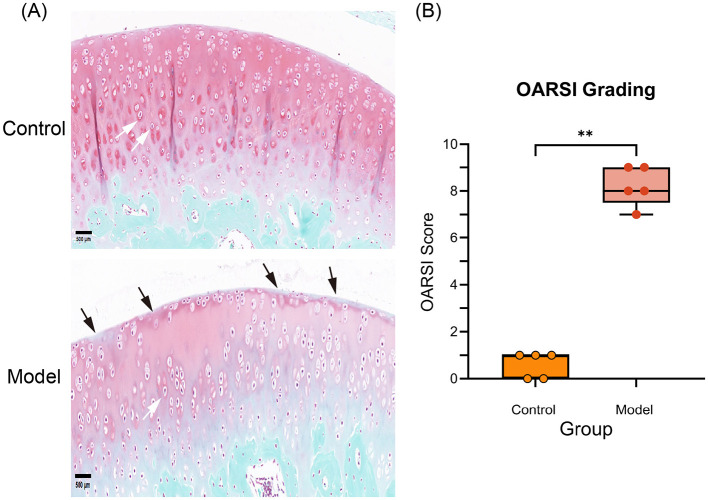
**(A)** Representative micrographs of the right knee joints of control and KOA rats 6 weeks after osteoarthritis induction, stained with safranin O (red) and fast green. Black arrows indicate cartilage surface wear on the femoral condyle, while white arrows indicate articular chondrocytes. Scale bar: 500 μm. **(B)** Mean OARSI scores for the right knee joint 6 weeks after OA induction in control and KOA model rats (*n* = 5 per group). **Indicates a statistically significant difference of *P* < 0.01 between the model group and the control group.

After 6 weeks, Safranin O/Fast Green staining revealed that control rat knees still had a smooth, undamaged articular surface with evenly stained matrix and regularly distributed chondrocytes. In contrast, cartilage from the model group showed surface fibrillation and proteoglycan depletion in the superficial zone, as indicated by decreased Safranin-O uptake. Chondrocytes seemed disordered, while the overall cartilage layer was noticeably thinner (Figure 5A). Quantitative OARSI scoring revealed significantly greater lesion grades in the model group compared to controls (*P* < 0.01; Figure 5B), indicating progressive osteoarthritic degeneration from joint immobility.

### 3.5 Apoptosis assessment in cartilage

**Figure 6:**
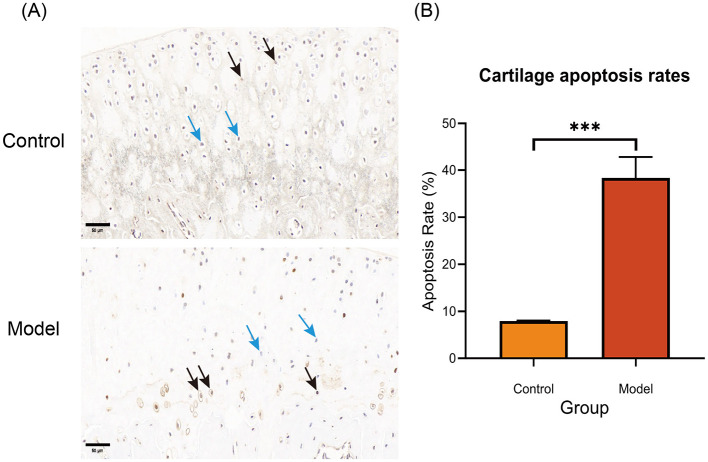
**(A)** Representative TUNEL-stained sections of articular cartilage from control and OA model rats. Black arrows denote TUNEL-positive (apoptotic) chondrocytes; blue arrows denote TUNEL-negative (viable) cells. Scale bar: 50 μm. **(B)** Quantification of chondrocyte apoptotic rates based on TUNEL staining (*n* = 3 per group). The OA model exhibited a significantly higher apoptotic rate than controls. ***Indicates a statistically significant difference of P < 0.001 between the model group and the control group.

TUNEL labeling was used to evaluate chondrocyte apoptosis. Six weeks after induction of the KOA rat model, the KOA group revealed a substantial increase in cartilage apoptosis compared to controls (38.36 ± 0.09% vs. 7.93 ± 4.43%; *P* < 0.001; Figures 6A, B). These findings were consistent with safranin O-fast green staining, which showed damage to the cartilage matrix and architecture in KOA rats. Overall, these findings support the effective creation of the rat KOA model and highlight the critical role of chondrocyte death in OA progression.

## 4 Discussion

In this study, we utilized a medical polyurethane composite bandage to immobilize the rat knee in full extension. This innovative method not only enhances the traditional Videman plaster model by improving ease of application and performance but also more accurately reproduces the long-term disuse pathology observed in the human knee joint (14). In comparison to conventional plaster casts, the polymer bandage offers rapid setting (1–2 min), reduced weight, significantly increased breathability (almost three times higher gas permeability), and adjustable stiffness. These characteristics collectively decrease skin ulceration and compression injury, leading to enhanced animal welfare and model reliability (17). To improve model stability and durability, we integrated a non-elastic secondary bandage beneath the polymer cast and included an anti-gnawing steel wire, resulting in a substantial reduction in complication rates. Multimodal assessments revealed that, over time, the knee cartilage surfaces of the model group exhibited roughness and irregularity upon gross inspection, and Modified Lequesne MG scores increased dramatically. Characteristic osteoarthritis alterations, including joint-space narrowing, subchondral sclerosis, and osteophyte formation, were validated using T_2_-weighted MRI and micro-CT imaging. TUNEL assays demonstrated an approximately 4.8-fold elevation in apoptotic chondrocytes compared to controls (*P* < 0.001), whereas Safranin O–Fast Green staining indicated proteoglycan depletion in the superficial cartilage layer, resulting in OARSI scores of 3 or higher. These diseased characteristics collectively resemble early human KOA closely. In conclusion, our combined imaging and histological data confirm the reliability of this rat model for mechanistic studies and therapeutic assessment, accurately reflecting joint-capsule laxity and uneven cartilage stress distribution observed in chronic disuse, thereby offering a solid foundation for exploring knee OA pathogenesis and interventions.

From the perspective of comparative and clinical medicine, the non-invasive rat KOA model established in this study demonstrates clear cross-species translational value for both veterinary and human health, with direct implications for clinical practice. (1) Cross-species Consistency: The Foundation for Comparative Medicine Applications. KOA pathological mechanisms and progression are highly conserved across humans, companion animals (dogs, cats), performance animals (horses), and rodents. Core pathological features—including progressive cartilage degradation, subchondral bone remodeling, synovial inflammation, and osteophyte formation—as well as key risk factors such as joint immobilization, trauma, aging, and obesity, are consistently observed across these species (7). Compared with murine and rabbit models, the Sprague-Dawley rat model used in this study offers distinct translational advantages. Mouse articular cartilage (30–50 μm thick) falls below the resolution limit of high-field MRI, impeding precise identification of lesions (18). Rabbit cartilage exhibits rapid *ex vivo* proteoglycan loss, is associated with higher feeding costs, and lacks species-specific antibody reagents (19). In contrast, rat knee articular cartilage (~0.1 mm thick) more closely resembles human and veterinary species in anatomical structure, biomechanical properties, and OA progression patterns, providing a solid scientific basis for cross-species data extrapolation (20). Importantly, the non-invasive immobilization model established in this study faithfully recapitulates the natural progressive course of OA, closely mirroring the clinical onset patterns of disuse-induced and post-traumatic OA in both humans and veterinary species. Unlike surgical and chemical induction models, this approach avoids confounding factors such as surgical trauma and excessive acute inflammation, offering greater cross-species clinical relevance and fully aligning with the preclinical research requirements of comparative medicine. (2) Specific Guidance for Veterinary and Human Clinical Practice. For veterinary health, this model directly addresses unmet clinical needs in the management of OA in companion and farm animals. It is well-suited for large-scale preclinical screening of analgesic and disease-modifying OA drugs for aged dogs and cats, as well as for evaluating preventive strategies in athletic horses and high-yield dairy cows to reduce economic losses and improve animal welfare (5). Furthermore, it provides a standardized tool for bidirectional translation of OA research between veterinary and human medicine, aligning with the core principles of the One Health initiative (21). For human health, this model offers a robust preclinical foundation for research into OA diagnosis, treatment, and prevention.

An effective animal model of OA must accurately mimic the disease's onset and progression. Although numerous models have been created, each possesses inherent limitations (10, 22, 23). The conventional Videman model, employing plaster immobilization in rabbits, frequently encounters issues including intricate application processes, challenges in removal, and a significant incidence of complications. In this study, we investigated these issues by utilizing Sprague-Dawley rats and immobilizing the knee joint with a medical polymer composite bandage. This method streamlined the process, improved model stability, and markedly decreased the likelihood of complications. Chemical induction models, such as intra-articular injections of sodium monoiodoacetate (MIA), are recognized for their swift induction of osteoarthritis-like symptoms. Nonetheless, they frequently provoke a significant inflammatory response that may obscure the initial degenerative alterations typical of human osteoarthritis (24). Our model induces chronic degeneration through the simulation of mechanical stress deprivation, which avoids acute inflammatory disturbances and more accurately reflects the natural progression of human osteoarthritis (25). Gu et al. (26) developed a rat KOA model through the modified Hulth method to examine alterations in miR-199-3p expression in cartilage (26). Surgical models, including anterior cruciate ligament transection, effectively induce osteoarthritis; however, they are invasive and may lead to considerable variability among subjects (27). The Sprague-Dawley rat model utilizing medical polymer composite bandage immobilization serves as a practical, reproducible, and physiologically relevant platform for investigating OA pathogenesis and assessing potential therapeutic interventions.

TUNEL labeling revealed a considerably higher chondrocyte apoptosis rate in the model group, implying that mechanical stress deprivation may cause mitochondria-dependent apoptosis by inhibiting the integrin-FAK signaling pathway (28). MRI scans revealed joint space narrowing, increased joint effusion, and a roughened cartilage surface following modeling, which is consistent with the degenerative characteristics seen in clinical KOA patients (29). Although inflammatory markers such as IL-1β were not directly evaluated, the observed pathological patterns indicate a positive feedback loop between inflammation and matrix breakdown in the degenerative process. Mechanistically, dynamic mechanical loading maintains cartilage matrix homeostasis maybe through TRPV4-mediated Ca2? signaling, regulating the expression of matrix synthesis genes (30, 31). On the other hand, Piezo1 channels may be activated by high mechanical stress, leading to ferroptosis and increased intracellular calcium influx. Piezo1 inhibition may increase chondrocyte viability (32). Mechanical signals may cause cartilage degradation by activating downstream pathways like Hippo-YAP/TAZ, NF-κB, and MAPK *via* integrins, ion channels, and the cytoskeleton (33–35).

We introduced four targeted refinements to the classic knee-immobilization protocol to maximize safety, stability, and user-friendliness. First, a non-elastic bandage is wrapped around the rat's knee before applying the polymer bandage, creating a soft interface that prevents thermal injury during curing, increases frictional grip, and markedly reduces animal struggling (and thus improves first-attempt success rates). Second, we replaced the bulky plaster of Paris with a medical polyurethane strip that sets in 1–2 min, requires only a single thin layer, and exerts minimal compression—cutting lower-limb swelling and necrosis rates from ~20 to ~2 % while maintaining excellent biocompatibility *in vivo*. Third, unlike rigid tube casts that necessitate power tools, our strips can be severed and removed in under 2 min with ordinary pliers, minimizing mechanical trauma at removal. Finally, a stainless-steel wire embedded within the softened polymer enhances overall stiffness and effectively deters gnawing, securing long-term fixation without additional restraint devices. Together, these optimizations yield an efficient, low-trauma rat KOA model with high reproducibility—ideal for extended mechanistic studies and preclinical therapeutic evaluations.

Despite its benefits, we have observed issues such as cast slippage, distal limb edema with occasional necrosis, and perineal irritation. To address these concerns and improve both the efficacy of the model and animal welfare, we have refined several key procedures. Firstly, we recommend thorough shaving 3–4 cm above and below the knee to ensure secure adhesion of the under-bandage and prevent slippage. Secondly, it is important to position the distal edge of the polymer strip just above the thigh root, fully covering the knee in a neutral (°) extension while avoiding contact with the perineal area to minimize soft-tissue irritation. Thirdly, from the application of the no-elastic bandage to the complete curing of the polymer bandage (1–2 min), it is crucial to maintain the knee at ° and wrap with consistent, moderate tension to ensure fixation without compromising circulation. Fourthly, we recommend using a “light wrap” during the initial week, applying minimal pressure to prevent slippage while closely monitoring distal perfusion, gradually increasing tension as the limb adjusts. Lastly, at the first sign of swelling, it is advised to promptly cut open the ankle strip, gently remove the bandage layer by layer to restore circulation, and re-immobilize once perfusion has normalized. These targeted refinements markedly reduce slippage, edema, necrosis, and infection, yielding a stable, reproducible rat KOA model suitable for long-term studies.

Similarly, this KOA rat model offers a versatile platform for evaluating both pharmacological and non-pharmacological interventions. For example, it can assess the efficacy of anti-apoptotic agents (e.g., caspase inhibitors) on chondrocyte apoptosis and associated signaling pathways (36–38). Furthermore, by adjusting the knee joint gap and joint mobility, the model may be utilized to evaluate the effectiveness and molecular foundation of reestablishing the mechanical balance of the joint in combination with traditional Chinese massage or joint release surgery. Additionally, the model is appropriate for physical factor treatment research, including the effects of microwave thermotherapy or therapeutic ultrasound on the expression of inflammatory factors and pain management (39–41). Since prolonged immobilization may induce muscle weakness and atrophy, this model can be used to explore strategies that prevent disuse changes (42). Additionally, it enables the evaluation of combined electroacupuncture or low-level laser therapy with exercise training for pain relief, functional improvement, and muscle preservation (43, 44). In summary, the KOA rat model established here not only provides a robust tool for dissecting pharmacological.

The non-invasive KOA model established in this study directly aligns with the core themes of diagnostics, therapeutics, and prevention in comparative and clinical medicine, offering clear translational value for both veterinary and human health. (1) For diagnostic research: this model faithfully recapitulates the full progressive pathological course of natural OA, making it well-suited for screening and validating early non-invasive diagnostic biomarkers—such as cartilage degradation metabolites and serum or synovial inflammatory factors. It thereby addresses a critical diagnostic gap for OA in both companion animals and humans. (2) For therapeutic research: with its advantages of non-invasiveness, high reproducibility, and low cost, this model provides an ideal platform for large-scale preclinical screening of disease-modifying OA drugs, rehabilitative therapies, and minimally invasive interventions. The resulting data offer a reliable foundation for subsequent veterinary and human clinical trials. (3) For preventive research: by inducing OA through disuse immobilization, this model accurately mimics clinical scenarios of disuse-induced OA—such as prolonged bed rest in humans or post-fracture immobilization in companion animals—thereby serving as a robust tool for evaluating the efficacy of OA preventive strategies. In summary, the rat KOA model established here not only provides a valuable tool for dissecting the mechanisms of both pharmacological and non-pharmacological interventions but also constitutes a reliable translational platform for advancing OA diagnostics, therapeutics, and prevention in human and veterinary medicine.

In this study, we established and systematically validated a novel non-invasive KOA model in Sprague-Dawley rats through behavioral, imaging, and histopathological evaluations, providing a robust preclinical platform for translational OA research in both human and veterinary comparative medicine. Despite these advancements, several limitations should be acknowledged. First, the sample size in this study was relatively modest. Future investigations will include larger cohorts to enhance the statistical power and robustness of the findings. Second, we did not perform a quantitative assessment of synovial inflammation. Subsequent validation will involve measuring key pro-inflammatory mediators, including IL-1β and TNF-α, to further characterize the inflammatory profile of this model (45, 46). Third, systematic biomechanical testing—such as cartilage stiffness measurement and joint biomechanical property analysis—was not conducted in the current study. These evaluations will be incorporated in follow-up research to comprehensively characterize the model and strengthen its translational validity. Fourth, although we compared the core characteristics of this model with mainstream preclinical KOA models through a literature review, head-to-head *in vivo* experimental comparisons with other established modeling methods were not performed to directly verify its relative advantages. These limitations will be systematically addressed in our subsequent studies to further optimize and validate this KOA model.

## 5 Conclusion

In conclusion, we have shown that using our approach, medical-grade polyurethane polymer bandages may be utilized to establish a reproducible, low-morbidity brake-induced OA model in rats, effectively replicating the cartilage thinning and subchondral alterations found with extended joint loading.

## Data Availability

The original contributions presented in the study are included in the article/supplementary material, further inquiries can be directed to the corresponding author.
